# Atomizing Instruments

**Published:** 1867-06

**Authors:** Benjamin Durham

**Affiliations:** Late Surgeon and Brevet Lieutenant-Colonel, U.S. Vols.


### ATOMIZING INSTRUMENTS.

BY BENJAMIN DURHAM, JR., M.D., late Surgeon and Brevet Lieutenant-Colonel, U.S. Vols.

In the medical profession there is a constant endeavor to apply remedial agents as directly to the diseased tissue as may be practicable, and in diseases of the throat and lungs, especially, inhalation, in some form, has long been practiced. An attempt has been made to introduce insufflation of dry powders, but practitioners have preferred to administer healing agents in the form of vapor, and inventive minds have brought forward apparatus to nebulize, atomize, or mechanically divide fluids into fine vapor. In 1858, Girons formed a spray by forcing liquids against a metallic plate, but soon after, Dr. Bergson, of Berlin, constructed an apparatus which is now generally used in the different patterns of atomizing instruments. Bergson's tubes are very simply formed, consisting of two cylinders, each drawn down to a capillary aperture at one end, and so placed at right angles to each other that the point of the perpendicular tube shall cover the lower half of the orifice of the horizontal tube. These can be made by any one, from glass julep tubes, and are often used by druggists in forming an aromatic spray, since, when arranged as above, and a current is driven through the horizontal tube, the air in the perpendicular tube is exhausted by suction, and if the lower extremity has been placed in any fluid, it will be drawn to the capillary orifice and there minutely divided by the cross-current, as a strong gust of wind will break into a fine spray the water falling from the conductor at the corner of a building. In order to produce a spray in internal cavities, as at the mouth of the uterus, the tubes have been so constructed as to be parallel with one another, and then one is bent at the capillary extremity into the proper relation to the other. Dr. W. B. Richardson's nebulizer is more complicated in its construction, and is described in the *Medical Record* for 1866, p. 243, but Bergson's tubes answer every practical purpose.

Having these tubes as the foundation of all atomizers, we find many mechanical appliances for furnishing the nebulizing current. The first and most simple is the breath of the operator, and the next is an India-rubber ball perforated at the base, so as to allow the entrance of air as soon as it is emptied. But in these cases, the vapor will be formed intermittingly, and the instrument now most commonly used is a rubber tube, terminating in two hollow balls placed at a short distance from one another; the lower being the hand-ball, which is used as in the common self-injecting syringes, and the upper one, (of thinner rubber, and covered with thinner netting to prevent undue distension,) being the regulator of the spray-producing current. This is an effective and simple instrument, but it has the disadvantage of so tiring the patient that generally it will be injurious for him to use it, or he will neglect the application of remedies on account of the consequent fatigue. And whenever the operator makes the applications himself, he soon finds the repeated closing of the hand very wearisome, and he wishes for some agent to produce the current necessary to form the spray. Dr. Siegle, of Stuttgart, constructed an instrument, in which steam was used in vaporizing, instead of air. In this country, similar apparatuses, with some modifications, have been manufactured by instrument-makers, but those of Codman and Shurtleff seem to be the most popular. In their machine, a sheet-iron stand supports a copper boiler, provided with a spring safety-valve and with an opening surrounded with rubber packing, through which a Bergson's tube is introduced, the other arm of the tube dipping into a cup containing the medicated fluid. After partially filling the boiler with water, steam is raised by the use of an alcohol lamp, and the current passing through the horizontal tube creates the vacuum, draws up the fluid, and forms the spray without inconvenience to the invalid, and, when once the instrument is well arranged, without the constant supervision of the physician. To prevent the vapor from reaching the patient's face and clothes, a movable stand is provided with a face-shield or glass funnel, which prevents the scattering of the spray and conducts it to the mouth, or, by attaching a flexible tube to the nose. Some of the steam will be condensed, and a cup receives the drippings from the large end of the funnel. Such an instrument costs twelve dollars, but a physician with some mechanical talent can provide himself with a less finished instrument at small expense.

Having described the mechanical appliances for atomizing, the question arises, what is the range of application and the value of this agency in the treatment of disease? The literature upon this subject has been limited to a few articles scattered through our medical journals, but this spring they announce the publication in London of "Inhalation as a means of local treatment of the organs of respiration by atomized fluids and gases," by Dr. Hermann Beigel, and in Philadelphia, a similar work by Dr. J. M. DaCosta, while a report on the "Therapeutics of Inhalation," is expected from Dr. J. S. Cohen at the next meeting of the American Medical Association.

We readily perceive the special application of inhalation of atomized fluids to diseases of the mouth and air-passages, yet we cannot rely on it in the treatment of these affections, but simply accept it as a valuable adjuvant to our therapeutic means. During high inflammatory action in the larynx or lungs, caution must be used in the inhalation of remedies, and mucilaginous or narcotic substances should be employed, for it is doubtful whether any benefit is experienced except the relief of the distress and coughing. It has been fully proved that the spray reaches the smaller tubes of the lungs when deeply breathed, and therefore the respiration should be quite superficial in diseases of the pharynx, but full and slow when it is desirable to reach the extreme parts of the lungs. The greatest benefit is found in treating the diseases of the pharyngeal cavity, because the remedies can be applied more directly and in greater quantity; but we will feel great satisfaction in the use of atomized fluids in chronic catarrhal affections of the larynx and in chronic bronchitis, and the putrid odor which sometimes accompanies the expectoration can be counteracted by the inhalation of astringents or balsams. In the early stages of phthisis it will be found of advantage in limiting the cough, dyspnœa, and expectoration, but all observers unite in affirming it will not stay the progress of the malady. In pulmonary hemorrhage, whether tubercular or not, it proves to be a decided increase to the forces previously at command, and if atomization were only valuable for applying styptics directly or near to the bleeding points, it should not be neglected. In nasal catarrh, by means of the tube affixed to the face-shield, the upper portion of the nose may be reached without the pain consequent on the use of injections, and patients breathe more freely, so that, experiencing less distress, they say the nose seems "cleared out."

In addition to this internal use of the atomized spray, it has been found advantageous in external applications to the eye, chronic ulcers, etc., and Cohen advocates its employment when it is desirable delicately and carefully to cauterize a diseased surface.

In regard to the medicaments that may be used with the atomizing instruments, we may mention, first, the vapor of water, either cool or hot, then any limpid effusion or decoction may be poured into the boiler instead of water, thus imparting to the current of steam remedial power, while any clear solution or fluid may be placed in the medicine cup, for, in practice, the articles of the materia medica which are changed in appearance by mingling with aqueous vapor seem to have the same therapeutic value as when unaltered, though we must, in the use of infusions, choose such as will not prove incompatible with the remedy which is atomized. When it is desirable, we can graduate the amount of fluid which may be atomized in any given time, by selecting Bergson's tubes with capillary orifices of the desired calibre, and it is well to have them of three sizes. But, it must be remembered, although only a limited per centum of the nebulized fluid passes into the lungs, yet absorption from the air-passages is very rapid, and we must use caution that too large a quantity of the remedy is not used by our patient.

The following remedies have been used with benefit in the atomizing instruments, *viz.*:—nitrate of silver, tincture of opium, morphine, atropine, extracts of hyoscyamus, belladonna, and conium, tannin, alum, acetate of lead, sulphate of zinc, iodine, bromine salts, permanganate of potash, bichloride of mercury, and *even* cod-liver oil. The sesquichloride and persulphate of iron prove invaluable styptics in hemorrhages, while some cases of asthma have been relieved by the arsenite of potash; Dr. Beigel introduced the use of the nitrate of aluminum in nervous affections of the trachea and larynx, and recommends limewater as a solvent of the diphtheritic membranes; glycerine is very soothing to the irritated air-passages, and destroys the fetor of some pharyngeal diseases; while chloride of sodium, at first theoretically employed to produce an artificial sea air, has proved to be beneficial in all stages of phthisis.

In conclusion, a few ordinary cases are appended as instances of the application of atomization. My first use of the steam atomizing instrument was personally, for severe inflammation of the left tonsil and side of the tongue. *Aug. 26, 1866*, the disease was fully developed, the jaw was firmly closed, and no application could be made topically. I resorted to atomization of a solution of tannin, seven grains to the ounce, and immediately experienced relief from pain and constrained breathing. The usual treatment by poulticing, inhaling vapor of hot vinegar, etc., would not relieve the distress, but sleep quickly followed the use of the steam-machine until the night of September 2d, when even atomization ceased to check the pain. The next morning, a large abscess burst, and no farther medication was required.

II. M. A. applied *Oct. 18th, 1866*, for advice concerning ozæna, which had been treated many months by various physicians, without benefit. After inhaling, through the nostril, a solution of iodine (two grains to the ounce), he expressed himself as relieved. After the previous use of injections he had experienced considerable suffering, but now he breathed freely, without pain. While under treatment he was benefited, but he was unwilling to take from his business the necessary time and attention to effect a cure.

III. L. M. applied *Dec. 17th, 1866*, for medicine for an annoying hacking cough, which had troubled her for two days. I found she had a slight attack of bronchitis, and prescribed the inhalation of a solution of morphine, one grain to the ounce. The next day she reported she had inhaled the medicine several times with marked relief, and needed no further medication.

IV. L. M. again applied *February 19th*, for a repetition of the same treatment which had previously benefited her. She had taken a hard cold, and was troubled with severe pharyngitis. I prescribed the inhalation of the solution of morphine, the next day employed a solution of tannin (eight grains to the ounce), and on the third found her convalescent and ready to acknowledge the comfort in using the inhalation.

V. J. D. was attacked with tonsillitis, *April 4th*, and used gargles of capsicum and whisky, vinegar, etc., without relief. I was called *April 5th*, and found him suffering pain, and very uneasy at his confinement. I prescribed the inhalation of the solution of tannin. As soon as he had once used the atomizing instrument, he expressed his opinion that it would help him, and promised to make repeated applications that day and night. The next mornng he was better, and discharged himself by going to his store and transacting his usual business.

